# The Peculiarities of Structure Formation and Properties of Zirconia-Based Nanocomposites with Addition of Al_2_O_3_ and NiO

**DOI:** 10.1186/s11671-017-1901-7

**Published:** 2017-02-17

**Authors:** I. Danilenko, G. Lasko, I. Brykhanova, V. Burkhovetski, L. Ahkhozov

**Affiliations:** 1 Donetsk Institute for Physics and Engineering named after O.O. Galkin NAS of Ukraine, Nauki av., 46, Kiev, 03680 Ukraine; 2 Institute for Materials Testing, Materials Science and Strength of Materials (IMWF), University of Stuttgart, Pfaffenwaldring 32, 70569 Stuttgart, Germany

**Keywords:** Zirconia composites, Fracture toughness, Phase transformation

## Abstract

The present study is devoted to the problem of enhancing fracture toughness of ZrO_2_ ceramic materials through the formation of composite structure by addition of Al_2_O_3_ and NiO particles. In this paper, we analyzed the general and distinguished features of microstructure of both composite materials and its effect on fracture toughness of materials. In this paper, we used the XRD, SEM, and EDS methods for determination of granulometric, phase, and chemical composition of sintered materials. The peculiarities of dependence of fracture toughness values from dopant concentration and changing the Y^3+^ amount in zirconia grains allow us to assume that at least two mechanisms can affect the fracture toughness of ZrO_2_ ceramics. Crack bridging/deflection processes with the “transformation toughening” affect the K_1C_ values depending on the dopant concentration. Crack deflection mechanism affects the K_1C_ values when the dopant concentrations are low, and transformation toughening affects the K_1C_ values when the dopant concentrations begin to have an impact on microstructure reorganization–redistribution of Y^3+^ ions and formation of Y^3+^-depleted grains with high ability to phase transformation.

## Background

Technical progress every year presents new and more stringent performance requirements for materials and devices. The lifetime and reliability of the devices should be increased, and the wear and fracture should be decreased significantly. This is especially true for durable ceramic products, operating in aggressive environments and at high temperatures, when the fracture of article can be initiated by small damage (pore, scratch, defects). This leads to formation of a new trend in material design—the production of materials tolerant to the defects. So, the structure of material, in our case ceramic material, must withstand external shocks. For example, these are self-healing materials [1, 2]. The corrosion resistance of silicon nitride ceramics can be increased by modifying its secondary phase. The formation of insoluble oxide layers will strongly reduce damage caused by subcritical crack growth. Creation of ceramic matrix composite (CMC) and metal matrix composite (MMC) materials also is the way of formation of the special microstructures in materials with enhanced properties. So, the development of new methods of formation of predetermined structure of ceramic material may improve the reliability of ceramic materials.

As it is known, the damage mechanisms depend on the structure and the type of the materials. Also, it is known that the hard materials tend to be brittle and materials with lower strength tend to be tougher. The damage process is associated with initiation and propagation of cracks in the material. As it was shown by Ritchie “the intrinsic damage processes that operate ahead of the tip of a crack to promote its propagation, and extrinsic crack-tip-shielding mechanisms that act mostly behind the crack tip to inhibit this propagation” [3]. Intrinsic toughening is the source of fracture resistance in ductile materials. This mechanism is effective against the initiation and propagation of cracks. Most metallic materials are toughened by this mechanism. Usually, brittle materials, such as ceramics, cannot be toughened by plastic deformation and have low values of fracture toughness. Typically, fracture toughness values for Al_2_O_3_, SiC, and Si_3_N_4_ are less than 3–4 MPa*m^1/2^. The extrinsic mechanisms, which are inherent to ceramic materials, are only effective in resisting crack propagation; they can have no effect on crack initiation. The basic variants of extrinsic toughening mechanisms are crack or fiber bridging and crack deflection.

Ceramic materials on the basis of zirconia are distinguished from other ceramic materials by the highest fracture toughness value because they demonstrate the well-known “transformation toughening effect,” which took part in the field of stresses caused by the propagation of cracks in sintered materials [4, 5]. The martensitic phase transformation (with increasing specific volume of transformed grains), which takes part in zirconia ceramic, is the manifestation of extrinsic toughening mechanism [6], but it happen ahead of the crack tip. So, zirconia-based materials have three variants of increasing the fracture toughness value—by transformation toughening, crack bridging, and crack deflection processes. Perhaps, the synergetic effects may be realized in the special composite structure.

There are many studies devoted to the formation of composite structures on the basis of zirconia. Zirconia–alumina (ZrO_2_–Al_2_O_3_) composites have been the subject of extensive research because they couple a high toughness with the desirable properties of alumina, i.e., good resistance to wear and chemical stability [7, 8]. Also, it has been reported that the addition of Al_2_O_3_ in a ZrO_2_ matrix can suppress the low-temperature degradation of mechanical properties of zirconia [9]. The addition of Al_2_O_3_, besides their low solubility in ZrO_2_, practically did not affect the phase stabilization, i.e., zirconia and alumina exist as separate phases [7, 10, 11]. But, our investigations [12] show that method of composite powder preparation has strong effect on fracture toughness value of material. It was shown that the increasing of K_1C_ value of zirconia ceramics with a small amount of alumina, sintered from nanopowders and obtained using co-precipitation technique, can be conditioned through a series of processes for composite structure formation during precipitation, crystallization, and sintering of composite nanopowders.

A small number of studies have been devoted to the influence of Ni and NiO particles on the fracture toughness of monolithic 3Y-TZP ceramic materials [13, 14]. Another widely known system on the basis of zirconia is 8YZrO_2_-Ni(NiO) system for SOFC anode [15]. The triple junction between zirconia and Ni particles ensues the high level of catalytic properties of porous ZrO_2_-Ni(NiO) composite material. As engineering material, the composite Al_2_O_3_–Ni is more studied. In the studies [16, 17], it was shown that addition of nickel inclusions in alumina and zirconia matrix leads to increase in the fracture toughness of alumina or zirconia ceramic material. In this work, it has found two facts which addition of NiO during sintering promotes to (i) stabilization of a cubic phase of zirconia and (ii) destabilization of tetragonal phase and a formation of monoclinic phase. The formation of monoclinic phase even at small quantities of NiO (0.3–2 wt%) leads to sample destruction. In our previous study [18], it was found that the phase transformation from tetragonal to monoclinic phase in 3Y-TZP-NiO composite occurs only during sintering in air environment, and during sintering in argon environment, there are no traces of monoclinic phase, but the fracture toughness value increased by 40–50% [19]. The increasing amount of cubic phase of zirconia was also found. Probably in these two cases [12, 19], we can see the synergetic effect—increasing transformability of zirconia T-phase, and crack deflection/bridging caused by appearing of zone of tensile or compressive stresses near the inclusions. In turn, inclusions are realized during creation and decomposition of solid solutions under sintering process, but the physical properties (coefficient of thermal expansion, Young’s modulus, etc.) of chosen dopants (Al_2_O_3_ and NiO) are quite different. In this study, we try to separate the influence of residual stresses and transformability of tetragonal phase on fracture toughness of zirconia-based composites. These effects cannot be realized without changes in microstructure and chemical and phase composition of matrix phase.

In this work, we try to analyze and summarize the facts of influence of the Al_2_O_3_ and NiO additions on the structure formation of structure of zirconia ceramic materials and linked these structure peculiarities with the fracture mechanisms of zirconia ceramics.

## Methods

### Material Synthesis

The matrix ZrO_2_-3 mol% Y_2_O_3_ nanopowders (3Y-ZrO_2_) and composite ZrO_2_-3 mol% Y_2_O_3_-Al_2_O_3_ (3Y-ZrO_2_–Al_2_O_3_) nanopowders were synthesized by a co-precipitation technique using ZrOCl_2_·nH_2_O and AlCl_3_·6H_2_O salts. Based on previous investigations [12], the amount of Al_2_O_3_ was 2 wt%. For understanding the trends of the other composition, the amounts of 0.5 and 1 wt% have been used if needed. All chemicals used were of chemical purity (SiO_2_ <0.008 wt%, Fe_2_O_3_ <0.01 wt%, Na_2_O <0.01 wt%). At first, the appropriate amounts of Y_2_O_3_ were dissolved in nitric acid; then, the zirconium and yttrium salts (in case of matrix material) and zirconium, aluminum, and yttrium salts (in case of composite material) were mixed with a propeller stirrer for 30 min and were subsequently added to an aqueous solution of the precipitant (25% NH_4_OH) with constant stirring. Sediments were mixed for 1 h at room temperature at a pH of 9. Sediments were then repeatedly washed and filtered with distilled water. Washing was carried out until a negative test for C1^−^ ions is obtained with the use of a silver nitrate solution. After washing and filtration, the hydrogel was dried in a microwave furnace with an output power of 700 W and at a frequency of 2.45 GHz. The calcination of dried zirconium hydroxides and composites was carried out in resistive furnaces at 700 °C with dwelling time 2 h. Because the nickel hydroxide is soluble in ammonia salts, the preparation of the nanocomposite ZrO_2_-3 mol% Y_2_O_3_-NiO (3Y-ZrO_2_-NiO) powders was conducted by mixing appropriate amounts of zirconia and nickel oxalate powders in distilled water using ultrasound at a frequency of 22 kHz. NiO in the composite nanopowders were obtained by the calcination of powders at 600 °C [fedor]. Based on the previous investigations [19], the amount of nickel oxide was 10 wt%, but for understanding the trends of the other composition, the amounts from 1 to 7.5 wt% have been used if needed.

Cylindrical (20-mm diameter and 3 mm in height) and rectangular (45 × 4 × 4 mm) specimens were prepared firstly by uniaxial cold pressing, then by isostatic pressing at 200 MPa, and finally by pressureless sintering at 1500 °C for 1 h in air atmosphere in case of ZrO_2_-3 mol% Y_2_O_3_ and ZrO_2_-3 mol% Y_2_O_3_ + Al_2_O_3_ and in argon atmosphere in case of ZrO_2_-3 mol% Y_2_O_3_-NiO. The sintering of ZrO_2_-3 mol% Y_2_O_3_-NiO composites was performed in argon atmosphere because the total sample destruction took place in case of sintering in air. The specimens used for mechanical testing were ground with a 180-grit diamond wheel and were subsequently polished with diamond slurries to minimize machining flaws.

### Material Characterization

The powders and sintered specimens were characterized by XRD (Dron-3) with Cu-Kα radiation for crystallite sizes and quantitative phase analyses by a proven method [20]. For identifying of the monoclinic (M), tetragonal (T), and cubic (C) phases of zirconia, as well as Ni, NiO, and Al_2_O_3_, the angular regions of 25°–45° and 71°–77° were used. Particle sizes of different calcined powders were estimated by transmission electron microscopy (TEM) (JEM 200, Jeol, Japan). Reliable data were obtained by analyzing data from 30 TEM fields.

The flexural strength was measured using a four-point bending test on polished samples with a cross-head speed of 0.5 mm/min (Tinius Olsen H50kT, USA). The inner and outer spans were 20 and 40 mm, respectively. The hardness and fracture toughness of the materials was measured at room temperature by the Vickers indentation technique (Vickers tester TP-7p-1) on mirror-polished surfaces with a 98- and 196-N load, respectively. At 196-N loads, the Palmquist type cracks were propagated in 3Y-TZP and composited with alumina. The fracture toughness values were calculated by Niihara equation for Palmquist type cracks [21]. The density was measured using the Archimedes method. The microstructures of the ceramics were studied by scanning electron microscopy (JSM 6490LV Jeol) of thermally etched surfaces at 1450 °C polished surfaces as well as fractured surfaces.

## Results and Discussion

### Powder Characterization

**Fig. 1:**
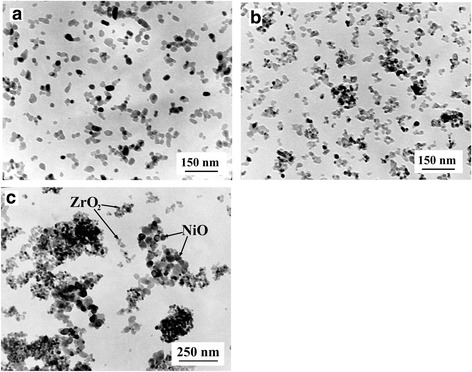
TEM structure of the oxide nanopowders. **a** 3Y-ZrO_2_ matrix. **b** 3Y-ZrO_2_-2 wt%Al_2_O_3_. **c** 3Y-ZrO_2_-10 wt%NiO

According to the electron microscopy and XRD data, the average particle size of matrix 3Y-TZP nanopowders was 17.5 nm. The mean particle size of ZrO_2_-3 mol% Y_2_O_3_-Al_2_O_3_ nanopowders, obtained by co-precipitation, decreased from 17.5 to 14.4 nm with increasing concentrations of Al_2_O_3_ from 0 to 2 wt%. After the calcination of the ZrO_2_-3 mol% Y_2_O_3_ + NiC_2_O_4_ nanopowder mix at 600 °C, nickel oxide was formed with an average particle size of 40 nm. All powders are represented in Fig. 1. Zirconia in powders was represented by the tetragonal phase (P4m2), and NiO was represented by the cubic phase (Fm3m). The absence of (101) Al_2_O_3_ peak at 43.36° in synthesized nanopowders and its appearance in the sintered material was discussed early [12].

In the case of ZrO_2_-3 mol% Y_2_O_3_-Al_2_O_3_ nanopowder, incorporation of Al^3+^ cations into the ZrO_2_ particles limited its crystallization [22] and consequently decreased the particle size of zirconia-alumina composite powders during calcination. According to ultrasonic mixing technology, the NiO NPs in zirconia matrix nanopowder can be distinguished by TEM (Fig. 1c) but not Al_2_O_3_ NPs (Fig. 1b).

### Characterization of Structure of Sintered Ceramic Materials

**Fig. 2:**
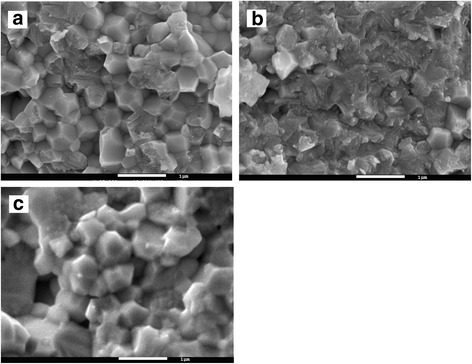
SEM microstructure of fractured surface of sintered materials. **a** 3Y-ZrO_2_ matrix. **b** 3Y-ZrO_2_-2 wt%Al_2_O_3_ composite. **c** 3Y-ZrO_2_-10 wt%NiO composite

After sintering at 1500 °C in air environment, the phase composition of zirconia in 3Y-ZrO_2_ matrix material and in 3Y-ZrO_2_-Al_2_O_3_ composites did not change according to XRD results. The phase composition was 9–11% in the cubic phase, with the remaining composition in the tetragonal phase. These structure parameters are typical for such chemical composition and sintering conditions. The SEM analysis of fracture surfaces of the samples has no differences in grain size of 3Y-ZrO_2_ matrix material and 3Y-ZrO_2_-Al_2_O_3_ composites, besides the character of fracture. In the case of 3Y-ZrO_2_ matrix material, the intercrystalline type of fracture was observed and in the case of 3Y-ZrO_2_-Al_2_O_3_ composites the transcrystalline one (Fig. 2). These peculiarities were studied in our previous work [12] and will be not discussed here.

**Fig. 3:**
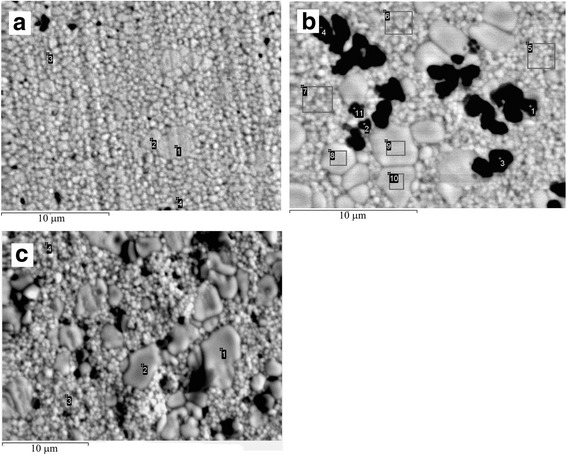
SEM microstructure thermally etched surface of 3Y-ZrO_2_ (**a**), 3Y-ZrO_2_-2 wt%Al_2_O_3_ composite material (**b**), and 3Y-ZrO_2_-10 wt%NiO composite material (**c**). Zirconia grains are *gray*, alumina grains are *black*, and NiO grains are *dark gray*

The average grain sizes with tetragonal and cubic phases in 3Y-ZrO_2_ matrix material by SEM data were 0.2–0.4 and 1–2 μm, respectively. In the case of 3Y-ZrO_2_-Al_2_O_3_ composite, the average grain sizes with tetragonal and cubic phases were 0.2–0.4 and 1– μm, respectively. But, the SEM analysis of thermally etched surfaces shows the macroscopic difference in the structure of zirconia grains between matrix material and composite (Fig. 3). Shown in Fig. 3b is the increasing number of “big” grains, which traditionally corresponds to the cubic phase of zirconia. The EDS analysis shows the increasing amount of Y^3+^ ions in the big grains, up to 8–10 wt% (4.5–5.5 mol%). This value is approaching the concentration of Y^3+^ ions, which corresponds to the chemical composition of cubic phase of ZrO_2_ (7–8 mol%), but it is not clear cubic phase. These data are coinciding with the data of Matsui [23], where it was showed that Al^3+^ ions segregated at grain boundaries directly enhance T → C phase transformation and grain growth at sintering temperatures above 1500 °C. The concentration of Y^3+^ ions in the matrix “small” grains of tetragonal phase decreased down to 2–2.5 mol% instead of 3 mol%, which corresponds to chemical composition of tetragonal phase of ZrO_2_. So, it was found that addition of Al_2_O_3_ to 3Y-ZrO_2_ ceramics leads to enrichment of some ZrO_2_ grains of tetragonal phase by Y^3+^ ions and depletion of the rest ZrO_2_ grains by Y^3+^ ions. Alumina in the sintered composites was represented by α-Al_2_O_3_ (black grains on Fig. 3b). The α-Al_2_O_3_ grains in 3Y-ZrO_2_-Al_2_O_3_ composites were presented as typical intercrystalline inclusions.

After sintering at 1500 °C in argon atmosphere, the phase composition of zirconia in 3Y-ZrO_2_ matrix material and 3Y-ZrO_2_-NiO composites changes according to XRD results. The amount of cubic phase in 3Y-ZrO_2_-NiO composite increased up to 20% in comparison with 3Y-ZrO_2_ (11%). SEM analysis of thermally etched surfaces also shows the macroscopic difference in the structure of zirconia grains. The amount of big grains, which traditionally corresponds to cubic phase of zirconia, and their size increased (Fig. 3c) in comparison with matrix 3Y-ZrO_2_. The average grain sizes with tetragonal and cubic phases in 3Y-ZrO_2_-NiO material by SEM data were 0.2–0.4 and 2–4 μm, respectively. The EDS analysis shows the increasing amount of Y^3+^ ions in the big grains up to 9–11 wt% (5–6 mol%). This value is approaching the concentration of Y^3+^ ions, which corresponds to chemical composition of cubic phase of ZrO_2_ (7–8 mol%). The concentration of Y^3+^ ions in the matrix small grains of tetragonal phase decreased to 1.6–2.5 mol% instead of 3 mol%, which corresponds to chemical composition of tetragonal phase of ZrO_2_. So, it was found that addition of NiO to 3Y-ZrO_2_ ceramics leads to depletion of ZrO_2_ grains of tetragonal phase by Y^3+^ ions, even more than addition of Al_2_O_3_.

### Mechanical Properties of Sintered Ceramic Materials

All samples were sintered to greater than 99% of theoretical density. The four-point bending strength values for 3Y-ZrO_2_-Al_2_O_3_ and 3Y-ZrO_2_-NiO composites decreased by less than 10% in comparison with 3Y-ZrO_2_ matrix material (from 850 ± 60 to 760 ± 70 and 820 ± 78 MPa, respectively). Hardness values for 3Y-ZrO_2_-Al_2_O_3_ composite increased slightly from 12.0 ± 0.2 to 12.45 ± 0.3 GPa and for 3Y-ZrO_2_-NiO composite from 12.0 ± 0.2 to 12.1 ± 0.3 GPa. We know that the absolute fracture toughness values obtained by the indentation method could be overestimated, but this technique has been approved by many authors to provide the estimation of the fracture toughness values for samples with high-density levels, where the porosity cannot have effect on crack propagation [11, 24–28].

**Fig. 4:**
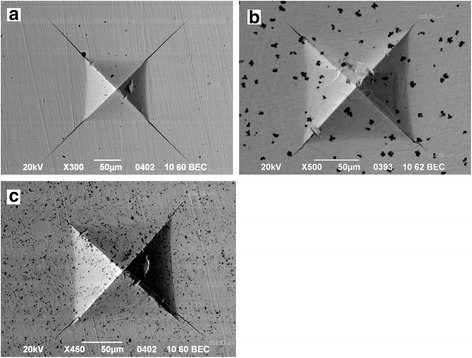
SEM images of Vickers indentation (196 N) and cracks at surface of sintered 3Y-ZrO_2_ matrix material (**a**), 3Y-ZrO_2_-2 wt%Al_2_O_3_ composite material (**b**), and 3Y-ZrO_2_—10 wt%NiO composite material (**c**)

**Fig. 5:**
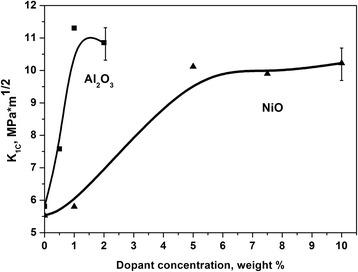
Dependence of indentation fracture toughness of 3Y-TZP-Al_2_O_3_ and 3Y-ZrO_2_-0 wt%NiO composite materials on Al_2_O_3_ and NiO content

Analysis of crack propagation after Vickers indentation showed that the crack length in matrix 3Y-ZrO_2_ material was 387 μm (Fig. 4a), in 3Y-ZrO_2_-2 wt%Al_2_O_3_ composite material was 208 μm, and in 3Y-ZrO_2_-10 wt%NiO composite was 237 μm (Fig. 4b, c). Decreasing the dopant concentration leads to increasing in the crack length and consequently to decreasing the fracture toughness value (Fig. 5)

Thus, it was found that increasing in fracture toughness value of zirconia-based ceramic composites by 40–50% in comparison with 3Y-ZrO_2_ is caused by addition of different types of dopants—Al_2_O_3_ and NiO. These materials strongly differ by crystal lattice (Al_2_O_3_—trigonal, NiO—cubic), density (Al_2_O_3_—3.96 g/cm^3^, NiO—7.45 g/cm^3^), CTE (Al_2_O_3_—8.86 × 10^−6^K^−1^, NiO—12.8 × 10^−6^K^−1^), and Young’s modulus (Al_2_O_3_—400 GPa, NiO—95 GPa). The analogical parameters for ZrO_2_ are 5.95–6.1 g/cm^3^ (density), 10.8–11.5 × 10^−6^ K^−1^ (CTE), and 195–205 GPa (Young’s modulus). So, besides the direct influence on crack propagation as inclusions of alien material, these inclusions affect the structure and phase composition of matrix zirconia, the distribution of residual stresses, and other physical and chemical properties. Let us consider the impact of these dopants on the fracture toughness of zirconia ceramics and try to find the general and distinguish features.

Analysis of the possible toughened mechanisms in 3Y-TZP-based ceramic composites shows that the basic toughening mechanisms are phase transition in zirconia, crack bridging, and deflection by inclusion grains. Let us start with the crack deflection and crack bridging processes which take place for different types of materials [3, 6, 11]. According to the equation from [11], the crack bridging by NiO grains can lead to increasing in K_1C_ value only by 0.1–0.3 MPa m^1/2^ and crack deflection by NiO grains on 0.6–1.0 MPa m^1/2^ in comparison with the matrix material ZrO_2_–3 mol%Y_2_O_3_. In the case of 3Y-ZrO_2_-Al_2_O_3_ composite, the increasing of K_1C_ value is even less, because the amount of Al_2_O_3_ is several times less than NiO. These calculations were performed in our previous works [12, 19]. For these calculations, the Young modulus for NiO was near 100 GPa [29] and for Al_2_O_3_ 380–400 GPa [30, 31]. The calculated fracture toughness increments explained the increasing of fracture toughness values due to the increasing number of filler grains (NiO or Al_2_O_3_) in composites (Fig. 5). The residual stresses around the inclusions have a great effect on the crack deflection. These stresses are conditioned with CTE mismatch between matrix material and inclusions. The calculation of the level of residual stresses (*q*) and its influence on fracture toughness values was done with the equation from Li [11] or Kern [28] studies,

1 $$ \varDelta K=2 q{\left(2\left(\lambda - d\right)\pi \right)}^2 $$

where *q* is the thermal residual stress in the matrix and *λ* is the average interparticle spacing, which can be related to the average diameter *d* and the volume fraction *f* of particles as follows:

2 $$ \lambda =1.085 d/{f}^{0.5} $$

and *q* can be calculated with the following equations [11]:

3 $$ q=-2 f\beta \varDelta \alpha {E}_m/ A $$

where *β* and *A* are the composition from the Young and Poisson modules of ZrO_2_ and Al_2_O_3_ or NiO [11].

**Fig. 6:**
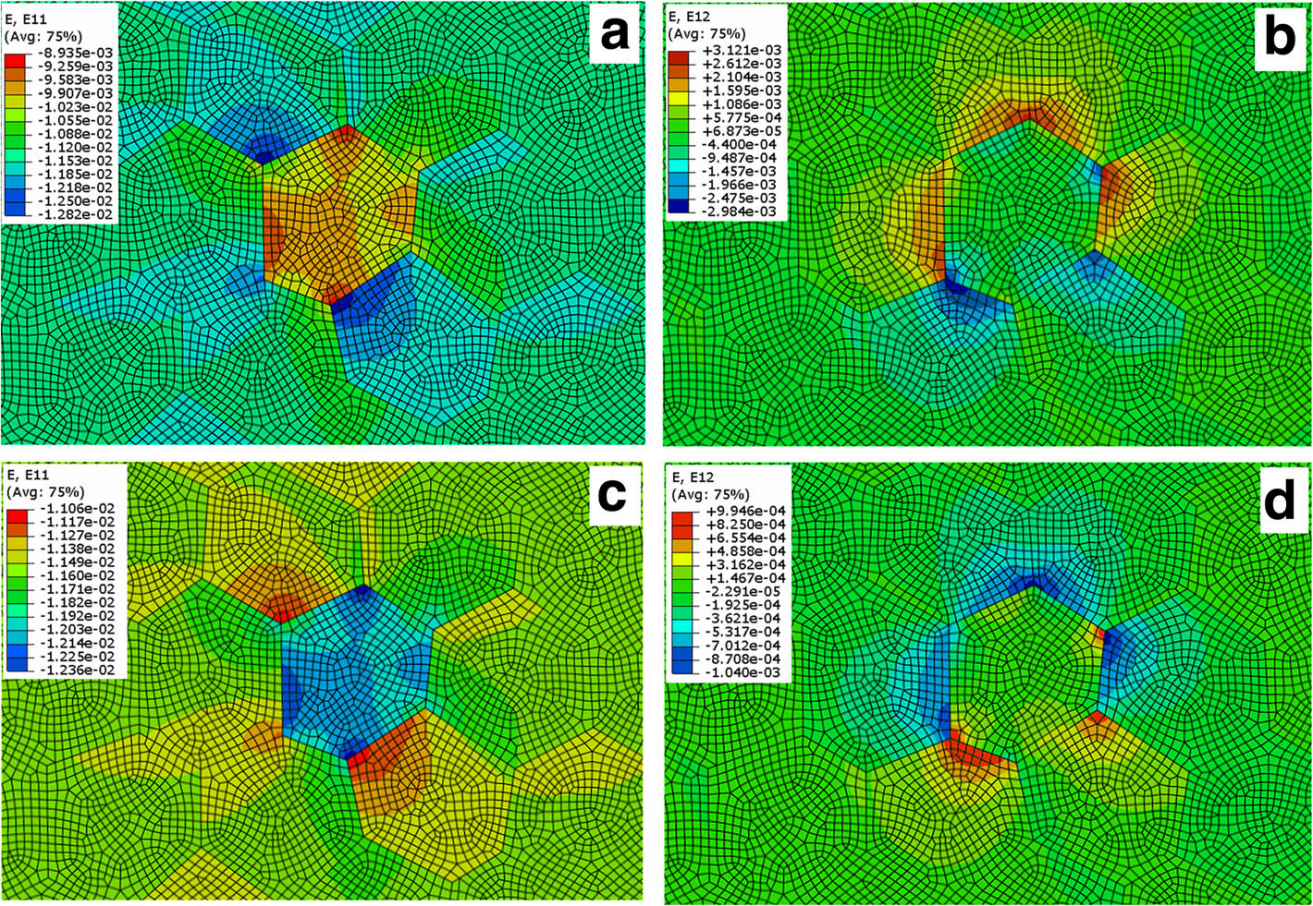
The calculated values of thermal normal (**a**, **c**) and tangential (**b**, **d**) residual stresses in and around Al_2_O_3_ (**a**, **b**) and NiO (**c**, **d**) inclusions in ZrO_2_ matrix. *Red color* compressive, *blue* color tensile stresses

The values of residual stresses around the second-phase particle are proportional to the differences in the thermal expansion coefficients between the matrix and the second-phase particle (*Δα*). Based on Timoshenko and Gudier theory and data from Awaji’s [32] work, we know that the stress state in the zirconia matrix with the Al_2_O_3_ second-phase inclusion is expressed as *σ*_*r*_ < 0 and *σ*_*θ*_ > 0 and matrix material near the boundary between matrix and inclusion is under radial compressive (*σ*_*r*_) and tensile tangential (*σ*_*θ*_) stresses. It is because the difference in CTE (*Δα = α*(ZrO_2_) − *α*(Al_2_O_3_)) between ZrO_2_ and Al_2_O_3_ is large enough (11.8 × 10^−6^–8.86 × 10^−6^ K^−1^). In the case of NiO inclusions, the difference in CTE values between ZrO_2_ and NiO decreases (11.8 × 10^−6^–12.86 × 10^−6^ K^−1^) but has an opposite (negative) sign. So, the stress state in the matrix material near the boundary between matrix and inclusion with the NiO second-phase inclusion is *σ*_*r*_ > 0 and *σ*_*θ*_ < 0; matrix material near NiO particles is under radial tensile stress and under compressive tangential stress. The computer simulation of the residual thermal stresses in these structures by ABAQUS software confirms these results (Fig. 6). This analysis shows that the residual stresses in the Al_2_O_3_ and NiO inclusions and around them are quite different and the crack tip behavior in these different conditions near the inclusion should be different too. For example, the crack may turn towards to the zone with high tensile stresses and it is the possibility of tensile fracture in brittle particles. Or, when the boundary between the matrix and the inclusion undergoes a highly compressive stress, this system is desirable for fabrication of dislocations around the dispersed particle, as it was shown in [32]. But, as mentioned above, the crack deflection and crack bridging processes that caused by intercrystalline type of NiO and Al_2_O_3_ inclusions cannot explain the high experimentally observed values of K_1C_. It is extremely pronounced in the case of Al_2_O_3_ inclusions, where the increasing of K_1C_ in 25% observed at 0.5 wt% of dopant. Also, it should be remembered that the enlarged amount of cubic phase in both types of composites may be a cause of slightly decreasing of fracture toughness value of samples, because the cubic phase has a lower indentation fracture toughness value in comparison with tetragonal phase.

The sharp increasing of fracture toughness may be conditioned only if we assume that the addition of NiO and Al_2_O_3_ affects the transformability of the tetragonal phase in 3Y-TZP. In our previous study [12], we investigate the crack propagation in 3Y-ZrO_2_-Al_2_O_3_ composite material and made a conclusion that formation of multi-level system of Al_2_O_3_ inclusions in combination with the enrichment of zirconia grain boundaries allows to increase fracture toughness of zirconia ceramics. But, detailed studies of SEM and EDS data from thermally etched surfaces of 3Y-ZrO_2_-Al_2_O_3_ composite material and comparison of these results with analogical data for 3Y-TZP-NiO (Fig. 3) allow us to suggest that the addition of Al_2_O_3_ and NiO promotes the increasing of Y^3+^ concentration in big zirconia grains in composites and leads to increasing metastability of rest Y^3+^-depleted ZrO_2_ grains. This correlation is shown in Table 1.

**Table 1:** The chemical composition on the polished and thermally etched surfaces of the grains of 3Y-ZrO_2_ matrix, 3Y-ZrO_2_-2 wt%Al_2_O_3_, and 3Y-ZrO_2_-10 wt%NiO composite materials. The numbers of spectrum are coinciding with the points marked on Fig. 3

Spectrum	Dopant	ZrO_2_	Y_2_O_3_	Note
	Without			
1	–	86.99	13.01	C-phase, Fig. 3a
2	–	87.16	12.84	C-phase, Fig. 3a
3	–	94.27	5.73	T-phase, Fig. 3a
4	–	94.60	5.40	T-phase, Fig. 3a
	Al_2_O_3_			
5	0.2	94.97	4.83	T-phase, Fig. 3b
6	0.12	94.65	5.23	T-phase, Fig. 3b
8	0.76	89.33	9.91	C-phase, Fig. 3b
9	0.46	88.97	10.57	C-phase, Fig. 3b
	NiO			
1	1.62	87.08	11.30	C-phase, Fig. 3c
2	2.02	86.37	11.61	C-phase, Fig. 3c
3	0.30	96.97	2.74	T-phase, Fig. 3c
4	0.98	94.92	4.11	T-phase, Fig. 3c

As it is known [33], this process took part in zirconia ceramic during sintering–cooling process. When the sintering temperature increases, the amount of segregated Y^3+^ ions on grain boundaries also increases because the diffusion process and ion segregation are enhanced, and a part of the tetragonal phase in Y-TZP transform into the cubic phase which is thermodynamically stable. During cooling, the grains, which were depleted by Y^3+^, become thermodynamically unstable and can be easily transformed into monoclinic phase in the stress field of propagated crack and stopped it. This is the transformation toughening effect [34]. In this case, the conception of critical grain size *d*_***c***_ is introduced. The grains with size smaller than *d*_***c***_ are stable, and grains with size greater than *d*_***c***_ are unstable and easily transformed into monoclinic phase. The *d*_***c***_ depends from dopant type and concentration and varied in a wide range from 150 to 1000 nm [34–36]. The most common value for tetragonal 3Y-ZrO_2_ is 300–400 nm. Increasing the dopant concentration leads to increase the critical grain size and, respectively, decreasing the dopant concentration leads to decreasing *d*_***c***_. The average grain size, which formed during sintering, depends on sintering conditions, initial powder characteristics, etc. For standard sintering conditions (1500 °C), which were used in this study, the average grain size is near 400 nm. So, decreasing the Y^3+^ ion concentration in zirconia grains in this study leads to decreasing the *d*_***c***_ to the value less then experimentally observed values of average grain size of ZrO_2_ in T-phase.

**Fig. 7:**
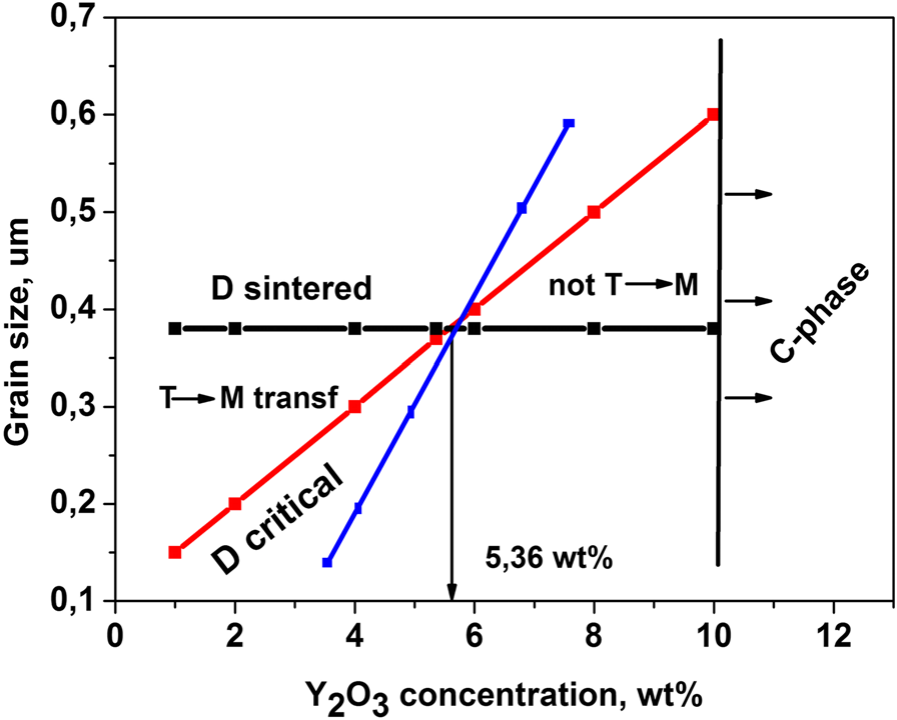
The scheme of transformability of ZrO_2_ grains from dopant concentration and grain size

As you can see from Table 1, the concentration Y^3+^ ions in small grains in both types of composites decreased faster in comparison with matrix 3Y-ZrO_2_ material. As mentioned above, the number and size of big zirconia grains with a high yttrium concentration in both composites are increased. In case of 3Y-ZrO_2_-NiO composite, these changes can be fixed by XRD. Because the experimental observed average size of zirconia grains in tetragonal phase in matrix 3Y-ZrO_2_ material and in both composites practically did not change but concentration of Y^3+^ in zirconia grains in T-phase in composites are decreased (Table 1), we can make a conclusion that the value of critical grain size of tetragonal phase in both composites are decreased (Fig. 7). The greater the influence of dopant ion on the phase stability, the faster the changes on the critical grain size is. Hence, the T-M phase transformation in Y^3+^-depleted grains can be realized more easily and transformation toughening effect may have more influence on fracture toughness value in both composites. The increasing of K_1C_ value with increasing dopant concentration and its stabilization after attaining the certain value of dopant concentration allows us to assume that at least two mechanisms can affect the fracture toughness of ZrO_2_ ceramics. First, mechanism is the crack bridging and deflection processes, which affects the K_1C_ value in a region where dopant concentration is low. In this region, K_1C_ values increases with increasing dopant concentration. Second, mechanism is the transformation toughening effect. It affects the K_1C_ values when the dopant concentrations increased to the critical values and can impact microstructure reorganization (formation of big grains with a higher Y^3+^ content). At low dopant concentration, this mechanism has low influence on K_1C_ value, but its influence increased with increasing dopant concentration. Dopants initiated the Y^3+^ ion diffusion in zirconia lattice and formation of Y^3+^ ion enrichment and depleted zirconia grains (formation big grains with higher Y^3+^ concentration). In this structure, the transformation toughening effect affects on fracture toughness value. The supersaturation of zirconia by Al_2_O_3_ and NiO dopants may reduce the K_1C_ values for the formation of zirconia grains with low stability (spontaneous transformation from tetragonal to monoclinic phase and damage of the samples due to cracking). By the way, we found in our previous studies a decrease of the K_1C_ value after the excess of the dopant concentration limits (5 wt% for Al_2_O_3_ and 20 wt% for NiO) [12, 19]. The interaction of these two mechanisms (alien grains in structure and phase destabilization in the matrix grains) can ensure the nonmonotonic dependence of K_1C_ value from dopant concentration. The differences in the “critical” concentration values (changing K_1C_ from increasing to stabilization) for Al_2_O_3_ and NiO may be explained as effect of dopant type and difference in the methods of powder synthesis (co-precipitation and mixing).

The key condition is that the dopant should enhance the diffusion of Y^3+^ ions in zirconia lattice and should not form the unwanted chemical compounds. By changing the type of dopant and its concentration, the formation of a ceramic material with enhanced level of fracture toughness and predetermined strength value can be created. So, the combination of structure peculiarities–multilevel inclusion structure and phase metastability can enhance the toughening mechanisms in zirconia-based composites.

## Conclusions

The effect of Al_2_O_3_ and NiO on microstructure peculiarities, mechanical properties, and fracture toughness behavior of 3Y-ZrO_2_ ceramics was investigated. The following conclusions could be drawn:

- By SEM data, it was shown that Al_2_O_3_ and NiO additions lead to acceleration of bimodal grain structure formation, when the Y^3+^ ion enrichment and depleted zirconia grains are formed.
- Analysis of influence of Al_2_O_3_ and NiO additions on indentation fracture toughness values of 3Y-ZrO_2_ ceramics shows the increasing of fracture toughness values with increasing dopant concentration and its stabilization on certain value.
- The combination of such behavior of K_1C_ dependences and structure peculiarities allows us to assume that at least two mechanisms can influence on fracture ZrO_2_ ceramics: (i) first mechanism is the crack bridging and deflection process, which affects in a region when K_1C_ grows with increasing dopant concentration; (ii) second mechanism is the transformation toughening effect, which exerts one’s influence with increasing dopant concentration to the highest level.
- The combination of multilevel inclusion structure and phase metastability can enhance the toughening mechanisms in zirconia-based composites.

## Abbreviations

3Y-ZrO_2_: 3 mol% yttria-stabilized tetragonal zirconia polycrystal
EDS: Energy-dispersive analysis
K_1C_: Critical coefficient of stress intension (K1)
SEM: Scanning electron microscopy
TEM: Transmission electron microscopy
XRD: X-ray diffraction
